# Monitoring of an Applied Beneficial *Trichoderma* Strain in Root-Associated Soil of Field-Grown Maize by MALDI-TOF MS

**DOI:** 10.3390/microorganisms11071655

**Published:** 2023-06-25

**Authors:** Thomas Edison E. dela Cruz, Jan Helge Behr, Joerg Geistlinger, Rita Grosch, Katja Witzel

**Affiliations:** 1 Department of Biological Sciences, College of Science, University of Santo Tomas (UST), España Blvd., Manila 1015, Philippines; 2 Department of Plant-Microbes Systems, Leibniz Institute of Vegetables and Ornamental Crops (IGZ), Theodor-Echtermeyer-Weg 1, 14979 Groβbeeren, Germany; behr@igzev.de (J.H.B.); grosch@igzev.de (R.G.); 3 Department of Agriculture, Landscape Development and Ecotrophology, Anhalt University of Applied Sciences (AUAS), Strenzfelder Allee 28, 06406 Bernburg, Germany; joerg.geistlinger@hs-anhalt.de

**Keywords:** bioinoculants, biomonitoring, field trials, protein profiling, soil fungi, sustainable agriculture

## Abstract

The persistence of beneficial microorganisms in the rhizosphere or surrounding soil following their application is a prerequisite for the effective interaction with the plant or indigenous microbial communities in the respective habitats. The goal of the study was to analyze the establishment and persistence of the applied beneficial *Trichoderma harzianum* (OMG16) strain in the maize root-associated soil depending on agricultural practice (soil management practice, N-fertilizer intensity) in a field experiment. A rapid identification of the inoculated strain OMG16 is essential for its monitoring. We used a culture-based approach coupled to matrix-assisted laser desorption ionization time-of-flight mass spectrometry (MALDI-TOF MS) analysis for the rapid identification of the inoculated *Trichoderma* strain as part of the beneficial microbe consortium (BMc). We isolated 428 fungal isolates from eight treatments of the field experiment. Forty eight percent of the isolated fungi equivalent to 205 fungal isolates were identified as *Trichoderma*, of which 87% (=179 isolates) were obtained from the fields inoculated with BMc. Gene sequence analysis showed a high similarity of the MALDI-TOF MS-identified *Trichoderma*, with that of the inoculated *Trichoderma harzianum* OMG16 confirming the re-isolation of the added beneficial fungus. This study highlighted the use of MALDI-TOF MS analysis as a quick, cost-effective detection and efficient monitoring tool for microbial-based bioinoculants in the field.

## 1. Introduction

The use of beneficial microorganisms as bioinoculants or biostimulants for plant or soil treatment can support sustainable plant production systems. Beneficial bacterial or fungal inoculants, often used either alone or in a consortium, have been reported to enhance overall plant growth, for instance, by increasing root growth through the production of phytohormones [1], by improving water and nutrient use efficiency of the plant [2,3], and by mitigating plant tolerance toward abiotic and biotic stresses [4,5]. The application of microbial-based biostimulants can be a part of an agriculture/horticulture management strategy for improving plant health and productivity.

Effects of microbial inoculants were often reported to be variable or lacking at field scale. Sufficient establishment and persistence of microbial inoculants in the rhizosphere or root-associated soil for an extended period were identified as prerequisites for their reliable activity in the interaction with the plant [6,7]. However, applied microbial inoculants have to compete with the indigenous microbial community in the soil or rhizosphere environment [8,9] and thus can have an influence on their establishment. The indigenous microbial community in the soil differs depending on the soil type [10] and is also affected by agricultural management practices [11]. Therefore, the ability of a beneficial microbial inoculant to establish in the soil/rhizosphere environment under field conditions was investigated.

Detection and quantification of microbial inoculants in the field can be achieved through culture-, microscopy-, and molecular-based methods [12], but each has its advantages and disadvantages. Culture-based methods were traditionally used for the isolation and selection of microorganisms, for instance, from environmental samples [13], and can be used for the re-isolation of an applied inoculant. In this case, a selectant with distinct antibiotic resistance must be generated from the microbial inoculant for its specific detection in environmental samples [14]. The generation of antibiotic-resistant selectants is more challenging for fungal than for bacterial strains. Methods for the genetic transformation of filamentous fungi, while available, require many steps, critical reagents, optimized protocols, and an extensively experienced researcher for a successful implementation, and for some methods, have low transformation frequencies [15]. Microscopy-based methods were also used for the monitoring of root colonization by microorganisms [16], and even for its quantification [17] but are not suitable for the identification of distinct strains in environmental samples.

PCR-based methods and next generation sequencing have all proven effective in examining the taxonomic composition of microbial communities, in the analysis of the relative abundance of distinct microbial taxa within microbial communities, in distinguishing microbial populations, and in observing shifts in microbial community composition and structure [18,19,20,21,22]. However, these methods are often costly and require robust bioinformatic analysis, and the effectivity depends on high-quality DNA extraction methods [23]. In addition, the detection of a specific microbial inoculant using PCR-based methods in environmental samples requires distinct sequence or genome information of the inoculated isolate. Sequence or genome information is used for the design of a specific primer pair that can be used for the specific detection of an inoculant.

In contrast to the mentioned methods, matrix-assisted laser desorption ionization time-of-flight mass spectrometry (MALDI-TOF MS) offers a rapid, cost-effective, and accurate approach for the specific identification of cultivable microorganisms based on their unique proteomic fingerprint. MALDI-TOF MS has been successfully applied in the diagnostics of microorganisms from clinical samples [24,25,26]. It has also been applied in environmental and food samples [27,28]. The successful application of MALDI-TOF MS profiling includes the detection of numerous biomarkers and its potential to capture and compare the organism’s fingerprints between normal and modified conditions [29] and in the addition of more reference mass spectra in corresponding databases [30]. MALDI-TOF MS has been successfully applied to characterize specific pathogenic *Fusarium* spp. [31] and *Trichoderma* spp. [32].

Fungal isolates, especially of the genus *Trichoderma*, are known for their ability to suppress soil-borne pathogens, for the induction of systemic plant defense responses, and for promoting growth in plants [33,34,35,36]. The goal of this study was to assess the establishment of the applied beneficial *T. harzianum* strain OMG16 in the root-associated soil of maize. *Trichoderma* is ubiquitous and exists in most soils, hence, the specific detection of the applied strain in environmental samples poses a challenge. For this purpose, we aim to evaluate whether the applied *Trichoderma* strain can be specifically detected in root-associated soil of maize by a combination of cultivation-based methods using a *Trichoderma* selective agar medium for isolation, and the MALDI-TOF MS analysis that allows for the specific identification of the inoculated *Trichoderma* strain. In case a specific detection is possible, we aim to answer the question whether agricultural farming practice affects the establishment of the applied *Trichoderma* strain. Previous studies showed that both the tillage practice and nitrogen (N) fertilization intensity affect the structure and composition of microbial communities in the soil and the applied strain has to compete with the respective microbial community.

## 2. Materials and Methods

### 2.1. Field Trials

The field trial was conducted in the frame of a long-term field experiment in Bernburg (Germany; 51.82° N, 11.70° E) in soil classified as loess chernozem over limestone (8% sand, 70% silt, 22% clay) with a neutral to slightly alkaline pH. Further site characteristics were previously published [37]. Maize plants (cv. Benedictio) were cultivated under two long-term soil tillage practices [mouldboard plough (MP) vs. cultivator tillage (CT)] and two N-fertilization intensities [intensive N-fertilization intensity with pesticide use (Int) vs. extensive N-fertilization (50%) without fungicide use (Ext)], with or without inoculation of a consortium (BMc) of three beneficial microorganisms *Pseudomonas* sp. (RU47; strain collection of the Julius Kühn-Institut, Braunschweig, Germany), *Bacillus atrophaeus* (ABi03; strain provided by ABiTEP GmbH, Berlin, Germany), and *Trichoderma harzianum* (OMG16; strain collection of Anhalt University of Applied Sciences, Bernburg, Germany). The microbial members of the consortium were selected based on their proven positive effects on plant health and performance [10,14,38,39]. Inoculation of these beneficial microorganisms (50 mL at a density of 2 × 10^8^ CFU/mL of each microorganism) occurred twice in an interval of 3 weeks during early plant developmental stages of maize, i.e., at week 3 for plant developmental stage (BBCH 13) and at week 6 after emergence (BBCH 31). Control plants were watered with 50 mL tap water. Root-associated soil from four replicates of each treatment (MP-Int, MP-Ext, CT-Int, CT-Ext, MP-Int+BMc, MP-Ext+BMc, CT-Int+BMc, CT-Ext+BMc) were sampled five weeks after the second inoculation at BBCH 65–69 for isolation of root-associated *Trichoderma* species.

### 2.2. Isolation of Trichoderma from Root-Associated Soil

**Figure 1:**
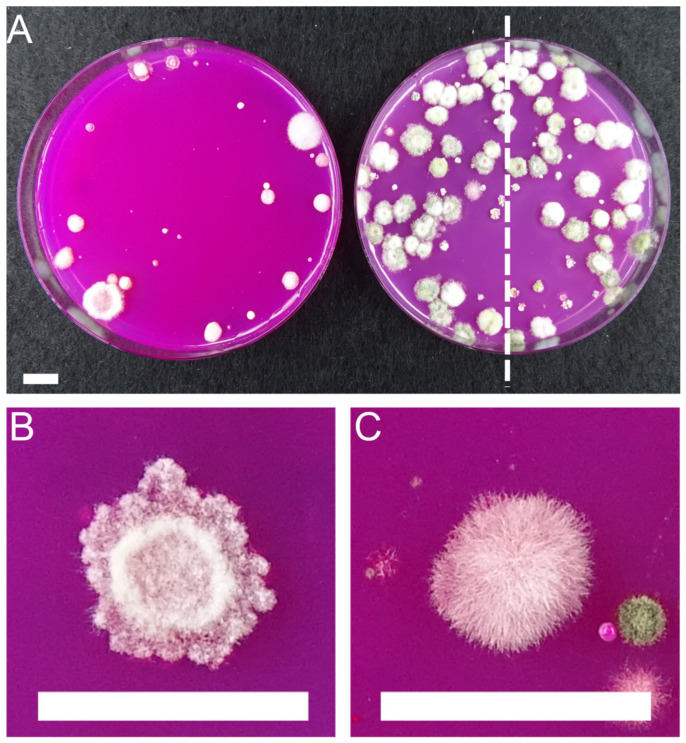
Colonies of root-associated fungi on Trichoderma selective medium (TSM). (**A**) Comparison of the colony density on TSM agar between extracts of control soil (left) and soil inoculated with BMc (right). (**B**) Phenotype of fungus presumptively identified as *Trichoderma* at CFU count. (**C**) Phenotype of fungus that was classified as non-*Trichoderma* at CFU count. Soil extract was diluted 1:10, plated on TSM and incubated for ten days at 25 °C in the dark. The dashed line indicates the halving of the plate at a high colony density for *Trichoderma* selection. Scale bars = 10 mm.

Loose soil adhering to the roots of the maize plants (five grams) was suspended in 50 mL saline solution (0.9% NaCl), diluted 1:10 and 1:100, and plated on *Trichoderma* selective medium (TSM), following Williams et al. [40], containing chloramphenicol (250 mg/L, Carl Roth, Karlsruhe, Germany), streptomycin (90 mg/L, Sigma-Aldrich, St. Louis, MO, USA), quintozene (200 mg/L, Sigma-Aldrich), propamocarb (930 mg/L, ProPlant, Arysta LifeScience, Paris, France) and rose Bengal (150 mg/L, AppliChem, Darmstadt, Germany). Following incubation at 25 °C for ten days, fungal colonies were counted at 1:10 dilution, the CFU per ml computed, and representative strains were isolated for species identification using MALDI-TOF (Table 1). Plates with a high CFU density of more than 30 colonies were halved in the middle, and colonies from one side were picked for identification (Figure 1).

### 2.3. Culture of Isolates and Extraction of Proteins

Root-associated fungal strains from the eight treatments, including four references of the inoculated *T. harzianum* OMG16 as control, were cultured on potato dextrose agar (PDA, Carl Roth) plates supplemented with 100 mg/L penicillin (Carl Roth), 50 mg/L streptomycin (Sigma-Aldrich), and 10 mg/L tetracycline (AppliChem). The four references of the inoculated *T. harzianum* OMG16 served as control; two references were obtained from the culture collection of the Anhalt University of Applied Science (AUAS) and two OMG16 isolates were re-isolated from the solution of the consortium with the beneficial microorganisms used for plant treatment. After an incubation time of 7–8 days at 25 °C, a small portion of the respective fungal mycelia of isolated strains and reference strains was transferred with an inoculating needle to Eppendorf tubes pre-filled with one mL potato dextrose broth (PDB, Carl Roth) supplemented with antibiotics (see above). The fungal cultures were incubated in a shaker at 25 °C for three days and afterward centrifuged at maximum speed for four minutes. The supernatant was carefully removed, 150 µL of LC-MS-grade water and 450 µL absolute ethanol was added to the pellet, and then centrifuged twice as above. The supernatant was discarded, and the fungal mycelial pellet was vacuum dried for ten minutes. Then, subsequently, 50 µL of 70% formic acid and 50 µL of acetonitrile was added to the pellet, vortexed, and centrifuged. The protein extracts were stored at −20 °C until use in the MALDI-TOF analysis.

### 2.4. Measurement of Protein Spectra by MALDI-TOF MS

Following the protocol of Djalali Farahani-Kofoet et al. [31] for the MALDI-TOF analysis, 1 µL of the protein extract was spotted on a polished steel target (Bruker Daltonik, Bremen, Germany) and allowed to dry. Then, 1 µL of the saturated HCCA (α-cyano-4-hydroxycinnamic acid solution) was added as matrix and allowed to dry. The MALDI method was calibrated using a bacterial test standard (Bruker Daltonik). The MALDI target plate containing the protein extracts was read with an ultrafleXtreme MALDI-TOF mass spectrometer (Bruker Daltonik) working in linear positive mode and acquiring mass spectra in the range of *m*/*z* 200–20,000. Measurements were performed by flexControl v3.4 software (Bruker Daltonik). The protein spectra served as the fingerprint of the test fungi and were compared with the reference database (1832 entries) of the Bruker Filamentous Fungi Library, including 13 entries for *Trichoderma* sp. The MALDI Biotyper v3.1 software (Bruker Daltonik) was used to process the raw spectra, generate peak lists and perform a database search for isolate identification. A dendrogram based on PCA clustering of *m*/*z* values was constructed using the hierarchical method, correlation distance measure, and average linkage algorithm.

### 2.5. Confirmation of Species Identity Using PCR-Based Method

Initially, we checked the specificity of the MALDI-TOF MS protein spectra by comparing 21 representative *Trichoderma* isolates from different treatments of the field experiment, including three reference strains of *T. harzianum* OMG16 and 12 isolates of *Fusarium* species, through principal component analysis. Then, the representative *Trichoderma* strains, as identified by MALDI-TOF MS, were initially grown on PDA and the mycelia were harvested and dried in liquid nitrogen. Genomic DNA extraction was performed with the EZNA Fungal DNA Mini-Kit (Omega Bio-Tek, Norcross, GA, USA) following the manufacturer’s instructions. DNA was amplified using PCR targeting the ITS region with the forward primer ITS1TrF (5′-ACTCCCAAACCCAATGTGAA-3′, Tm: 53.9 °C) and reverse primer ITS4TrR (5′-TGTGCAAACTACTGCGCA-3′, Tm: 54.6 °C) as described previously by Meincke et al. [41]. The PCR conditions were as follows: initial step at 94 °C (5 min), 35 cycles of 94 °C (30 s), 53 °C (35 s), and 72 °C (2 min), followed by a final extension step at 72 °C (10 min). The forward sequences were then compared to other available sequences in GenBank using the BLAST tool to obtain preliminary identities. Then, the sequences of the representative *T. harzianum* strains including ex-living type (CBS 226.95) were obtained from Gu et al. [42] and multiple alignments were estimated using ClustalW in MEGA v.11 [43] and manually adjusted when necessary. The best fit substitution model was also determined as Tamura 3-parameter (T92) + gamma distribution (G). Phylogenetic relationships based on nucleotide sequence similarity were inferred using maximum likelihood in 500 bootstrap replicated runs.

## 3. Results

### 3.1. Agriculture Practice Affect Presence of Trichoderma Strains in Root-Associated Soil

A total of 428 protein mass spectra from 428 fungal isolates were generated in this study. Of these, 205 (or 48%) of the isolated fungi were identified as belonging to the genus *Trichoderma* (Table 2). Most *Trichoderma* isolates were obtained from the treatments inoculated with the BMc that included the *T. harzianum* OMG16 strain. This observation indicates that the applied *Trichoderma* strain established in the root-associated soil of the respective treatments. The non-*Trichoderma* isolates matched the mass protein spectra of other fungal genera. Identification of the *Trichoderma* isolates was supported by the visual observation of their colonial growth on PDA (Figure 1).

A higher number of *Trichoderma* isolates was observed in the root-associated soil of maize when grown under long-term MP practice (117 isolates) compared to CT practice (88 isolates). Extensive or reduced N-fertilization (116 isolates) seemed to favor the presence of *Trichoderma* in the root-associated soil as compared to intensive N-fertilization intensity (89 isolates). Based on the high number of *Trichoderma* isolates in the consortium-inoculated treatments, it can be concluded that the added beneficial *Trichoderma* remained in the root-associated soil during the growing period of maize.

### 3.2. Species Identification of Trichoderma Strains

**Figure 2:**
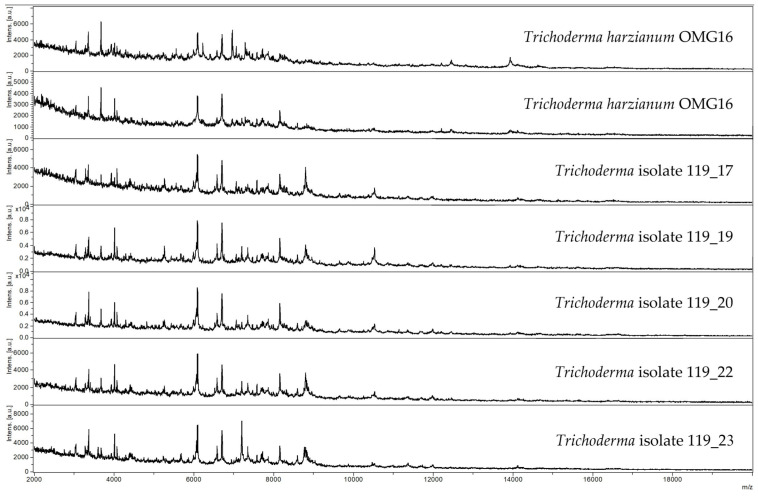
Protein mass spectra of the isolated *Trichoderma* strains from MALDI-TOF MS analysis. The first two spectra represent the *T. harzianum* OMG16 reference strains, while the remaining five spectra correspond to presumptive *Trichoderma* isolates 119_17, 119_19, 119_20, 119_22, and 119_23. These fungal strains were isolated from root-associated soil with the consortium of beneficial microorganisms inoculated onto maize plants (cv. Benedictio) grown in soil under moldboard plough and extensive N-fertilization intensity without fungicide use (MP-Ext+BMc).

**Figure 3:**
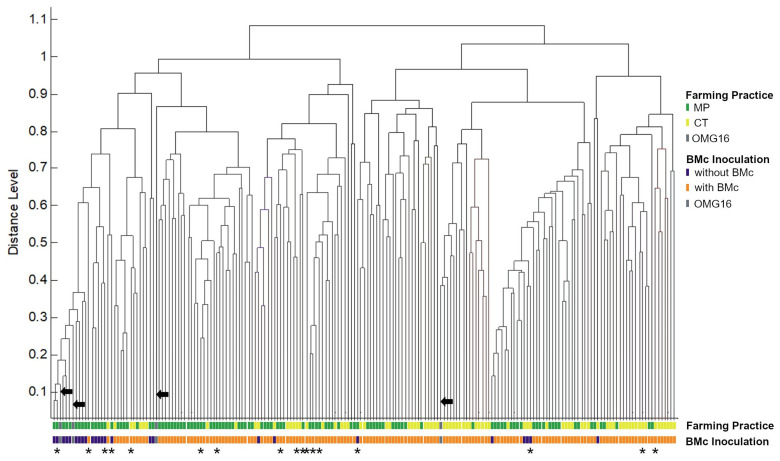
PCA dendrogram of 195 *Trichoderma* strains. The four reference strains of *T. harzianum* OMG16 are indicated by arrows while asterisks represent the *Trichoderma* isolates that were confirmed by gene sequence analysis (see also Figure 5). The Trichoderma isolates were further categorized based on farming practices [moldboard plough (MP) vs. cultivator tillage (CT)] with or without consortium of beneficial microbes (+BMc).

Species identification with MALDI-TOF MS requires a comparison of the protein mass spectra fingerprint of the isolates with the reference database. In this study, the protein mass spectra were compared against the Bruker Filamentous Fungi Library and matched with the spectra of mainly *Trichoderma* sp. CBS124027 (204 isolates) and *Trichoderma hamatum* CBS123063 (1 isolate). A comparison of CBS124027 with the fungal collection and database of the Westerdijk Fungal Biodiversity Institute, formerly Centraalbureau voor Schimmelcultures (CBS), matched with *T. harzianum*, a soil fungus isolated from Peru. In addition, MALDI-TOF MS analysis enables the comparison and grouping of spectra even without successful identification to identify recurrent isolated fungal strains. The number of *Trichoderma* isolates per match log score was as follows: >2.00 (2 isolates), 1.70–2.00 (60 isolates), and <1.70 (143 isolates). Furthermore, the protein mass spectra of the *Trichoderma* isolates clearly matched those of the control, albeit differences in peak heights were observed in some isolates (Figure 2). We then compared the protein spectra of 191 MALDI-TOF-identified *Trichoderma* selected from the 205 isolates and the four reference cultures of *T. harzianum* OMG16, i.e., two spectra from OMG16 strains inoculated during the field trials and two spectra from the original, preserved OMG16 cultures (see Section 2.3) by principal component analysis (PCA)-based clustering. Clearly, the added beneficial *Trichoderma* OMG16 grouped within the isolated *Trichoderma* isolates, indicating high similarities in their protein mass spectra (Figure 3). *Trichoderma* isolated from treatments without added BMc generally clustered together; those occasional isolates grouped within clusters of *Trichoderma* from BMc-inoculated field trials. We did not observe clustering based on farming practice.

**Figure 4:**
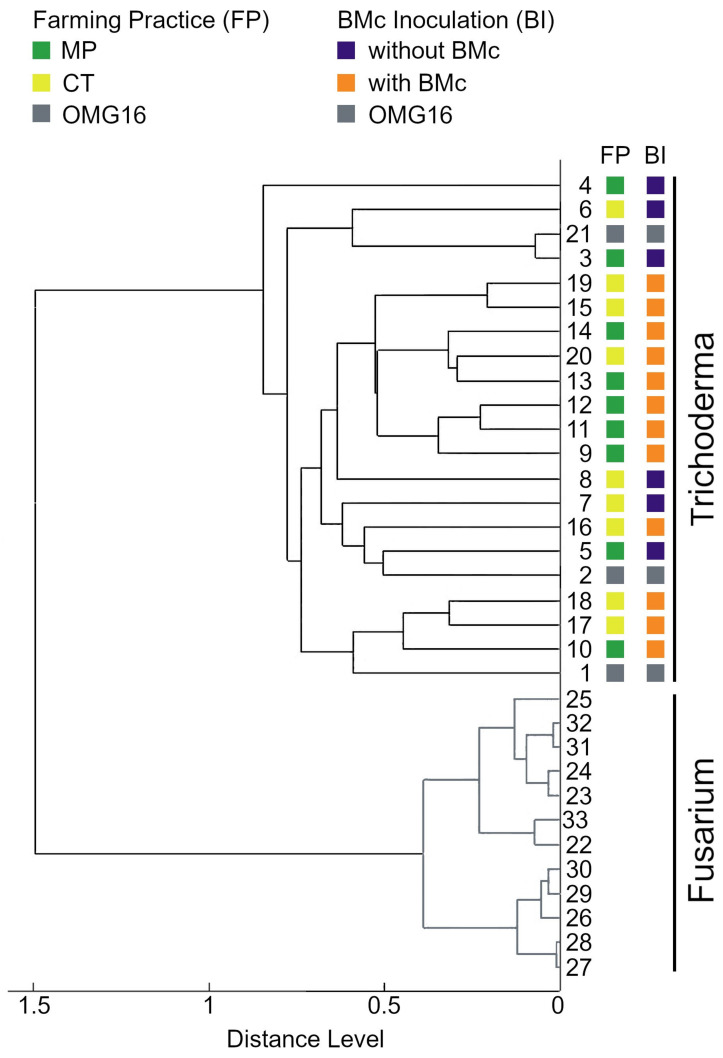
PCA dendrogram based on protein profiles of 21 *Trichoderma* strains from eight field trial treatments, including three reference strains of *T. harzianum* OMG16. The field trial treatments were based on farming practices [moldboard plough (MP) vs. cultivator tillage (CT)] with or without a consortium of beneficial microbes (+BMc).

To check the specificity of the protein spectra in identifying our target fungi, we compared the profiles of 21 representative *Trichoderma* strains from each of our field trials, including the reference strains of *T. harzianum* OMG16, with the protein spectra of twelve isolates of *Fusarium* species by PCA-based clustering. The *Fusarium* isolates clearly formed a cluster distinct from that of the *Trichoderma* (Figure 4). The reference strains grouped also within the isolated *Trichoderma*. Except for the three isolates from field trials that did not receive BMc, all *Trichoderma* isolates from BMc-treated soil formed a subcluster, indicating very high similarities between their protein spectra. However, we did not see any influence of the agricultural practice (tillage, N-fertilization intensity) on the protein patterns of the different *Trichoderma* isolates.

**Figure 5:**
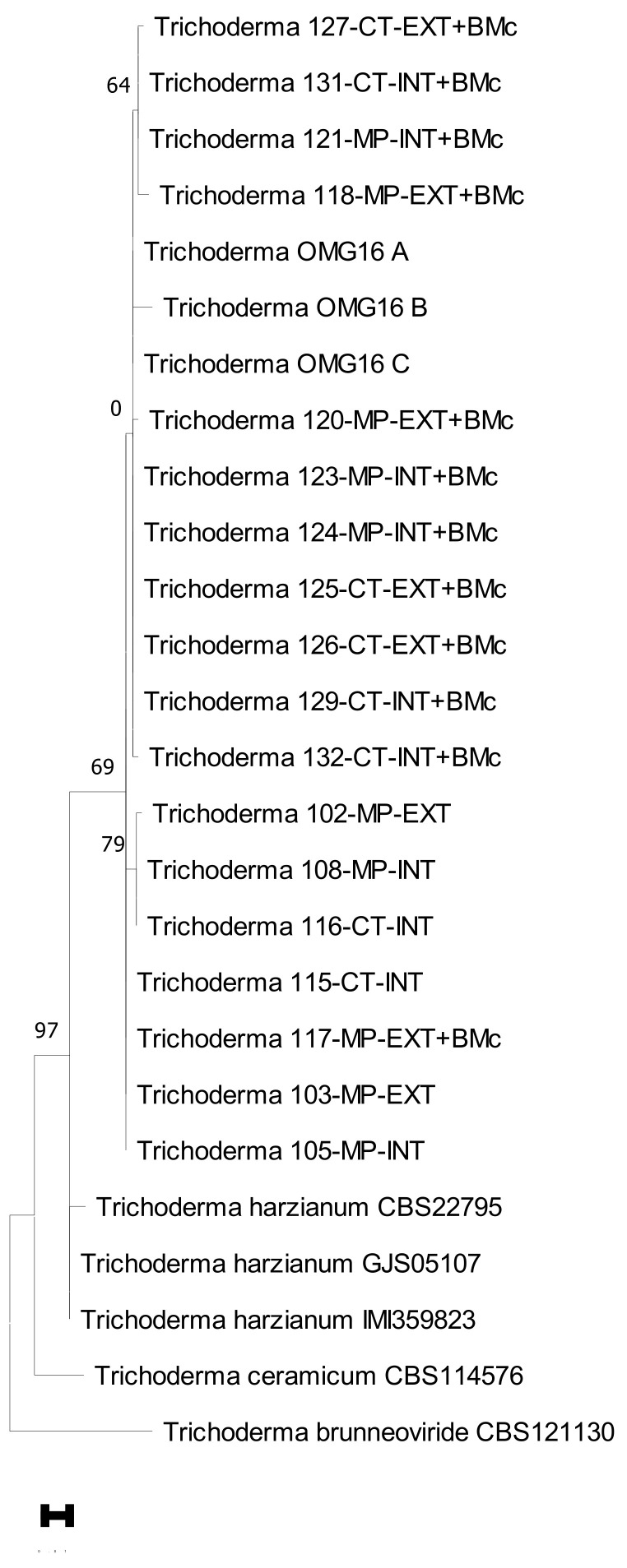
Phylogram showing the 21 representative *Trichoderma* isolates with three reference strains of *T. harzianum* OMG16 (control) and other *T. harzianum* strains. Isolate number includes the tillage practice [moldboard plough (MP) vs. cultivator tillage (CT)] and N-fertilization intensity [intensive with pesticide use (Int) vs. extensive without fungicide use (Ext)] with or without the consortium of beneficial microbes (+BMc).

To further confirm whether the *Trichoderma* as identified by the MALDI analysis were either comparable to or the same as the added beneficial *T. harzianum* OMG16 isolate, the 21 fungal strains representing the different field treatments were subjected to a gene sequence analysis of the ITS gene regions (Figure 5). Our phylogram clearly confirmed the identities of our isolates as it showed a high bootstrap support (97%) with other strains of *T. harzianum*. The OMG16 strains also clearly grouped within our identified *Trichoderma*. Of the 446 aligned ITS bases, the 21 isolates differed from each other by only 29 bases. The OMG16 strains differed by only 14 bases from the isolates from BMc-treated soil as opposed to a 26-bases difference with those isolates from non-BMc soil.

## 4. Discussion

*Trichoderma* spp. are known mainly as soil-borne ascomycetous fungi that are present in a wide range of geographical locations. Species of this genus have been isolated from various ecological sources such as soil, water, and plant habitats, including the rhizosphere and roots of associated plants [35,44,45,46,47,48]. This indicates the ubiquitous nature of the genus *Trichoderma*. The genus also includes a number of beneficial species known to act as plant growth stimulants through, for instance, increases in the lateral and primary root lengths that influence the effectiveness of nutrient uptake by the plant [49] or through the production of a large number of secondary metabolites and creating a favorable environment [50]. Most studies demonstrated that beneficial *Trichoderma* improves overall plant health and has great potential to support sustainable plant production systems. However, for successful interaction with the plant, beneficial *Trichoderma* isolates must establish in the rhizosphere or root-associated soil. The detection and monitoring of microbial inoculants in soil and the analysis of their fate and persistence are the major issues for bioinoculants to be implemented in agricultural practice [51]. The very similar phenotype of *Trichoderma* species in soils makes it difficult to identify a distinct strain in environmental samples.

In this study, we combined a culture-dependent approach using a selective medium for *Trichoderma* isolates from root-associated soil with MALDI-TOF analysis for the identification of the obtained isolates, including the specific detection of the applied OMG16 strain. Based on the combined approaches, we aimed to find out whether the applied *T. harzianum* strain OMG16 can be specifically detected in the samples of root-associated soil of maize during the growing period. The results provided information on the establishment of the applied strain in this habitat and its establishment in differing tillage practice and N-fertilization intensity. *Trichoderma* isolates could be successfully re-isolated from all environmental samples by the used *Trichoderma* selective culture medium. This selective medium is also designed for the quantification of *Trichoderma* species from soil samples owing to the added bacterial and fungal inhibitors and low concentration of glucose [52] as performed in this study. However, while this medium was designed to be selective for *Trichoderma*, and thus, expected to facilitate only the growth of *Trichoderma*, it is not entirely capable to inhibit the growth of other fungi as found here. This poses a challenge of visually differentiating colonies of *Trichoderma* from non-*Trichoderma*. Interestingly, nearly twice the number of other fungal taxa were found in the treatments without inoculation of the consortium of beneficial microorganisms (BMc), including the *T. harzianum* strain OMG16, while about seven times more *Trichoderma* isolates were isolated from the treatments inoculated with the BMc (Table 2). This indicates that the applied *T. harzianum* strain OMG16 established in root-associated soil of maize. The *Trichoderma*-selective culture medium, which favors the growth of *Trichoderma* while inhibiting non-targeted fungi and bacteria, is thus recommended for the isolation of *Trichoderma* from environmental samples. Visual observation such as the mode of mycelial growth, color, arrangement, and the development of conidiophores can support the identification of *Trichoderma* isolates. In this study, MALDI-TOF MS was used for the specific and quick identification of the applied *Trichoderma* strain by comparing the protein mass spectra fingerprints of the isolated *Trichoderma* strains with that of the applied OMG16 strain. MALDI-TOF analysis can provide an accurate identification of fungal species and represents a cost-effective, quick, and easy-to-use tool for the identification of fungal isolates. Protein extraction, MALDI-TOF MS processing, and data, analysis for species identification via database comparison could be performed in an hourly range, depending on the number of samples (Table 3). Even high numbers of samples can be analyzed within a short time; therefore, besides species identification, the tool can be used for the continuous monitoring of an applied fungal isolate in environmental samples, for instance, throughout field experiments. In this study, protein mass spectra fingerprints of 191 *Trichoderma* isolates were comparable to the applied *T. harzianum* strain OMG16. This result confirmed the establishment in the root-associated soil of maize. Although we isolated *Trichoderma* from different farming practices and fertilization regimes with or without BMc inoculation, we did not observe any clear influence of these agricultural practices on the root-associated *Trichoderma*.

The use of PCR-based methods has successfully traced and discriminated non-native isolates of arbuscular mycorrhizal fungi from the field up to 2 years post-inoculation [53]. With the advent of modern technology, a qPCR assay that targets the RNA polymerase II gene (RPB1) quantitatively traced the non-native arbuscular mycorrhizal fungus *Rhizophagus irregularis* isolate IR27 from the native *R. irregularis* isolates in roots [54]. While PCR-based methods have been proven successful in discriminating native and non-native fungal strains, other microbial quantification methods can also be applied, particularly when issues such as budgetary constraints are taken into account. For example, culture-based methods with enrichment, selective, or differential media, particularly the use of synthetic minimal medium, can be easily conducted to re-isolate the added bioinoculant, as shown with the use of a *Trichoderma* selective medium in this study. However, the isolated bioinoculant must still be identified, as well as with PCR-based methods such as DNA barcoding, which could add to laboratory expenses. DNA barcoding also necessitates the sequencing of target genes, which, if conducted through outsourced service, may also take a considerable amount of time. Here, we proposed the use of MALDI-TOF as a cost-effective, timesaving, and easy-to-use tool for the identification of re-isolated bioinoculants and its continuous monitoring throughout field experiments or trials. Excluding the initial culture of the test fungus, which could last up to three days to a few weeks depending on the species, the extraction of the proteins and MALDI-TOF MS processing, up to the data analysis of species identification via database comparison could last only for a few hours, depending on the number of samples. Species identification with MALDI-TOF MS requires a comparison of the protein mass spectra fingerprint of the isolates with the reference database and can also be performed with other fungal isolates (Figure 4). This provides an accurate identification of species. For the tracking and monitoring of specific bioinoculants, identification can also be accomplished through a comparison of the protein spectra with controls, i.e., the specific strain of the added microbial inoculum as shown in this study. Figure 4 and Figure 5 show the hierarchical clustering based on the protein fingerprint of our microbial inoculant, *T. harzianum* OMG16, with our re-isolated *Trichoderma* strains, and the confirmation of its identity through gene sequencing.

In summary, culture-based approaches for the isolation of bioinoculants are time-consuming, labor-extensive, and require expertise in correct species identification using colonial and morphological characters. If used together with PCR-based methods, i.e., species identification via DNA barcoding, the approach will necessitate a considerable amount of time and money for genomic DNA extraction, PCR amplification, sequencing, and bioinformatic analysis. However, a culture-based approach coupled with MALDI-TOF MS analysis for species identification is an easy and quick (short turn-around time) method that does not require extensive training on the use of the equipment. As such, it can be easily employed for the effective tracking and monitoring of bioinoculants in field trials where successful establishment is required and during real-life applications of beneficial microbes. While we recognize that PCR-based methods offer a more accurate approach to species identification, our method has an advantage of being less costly, particularly when testing large numbers of samples, as shown in our study.

## Figures and Tables

**Table 1:** Number of fungal isolates and the colony-forming unit (CFU) count per treatment depending on tillage practice [moldboard plough (MP) vs. cultivator tillage (CT)] and N-fertilization intensity [intensive N-fertilization intensity with pesticide use (Int) vs. extensive N-fertilization (50%) without fungicide use (Ext)] and inoculation of a consortium (BMc) with beneficial microorganisms [*Pseudomonas* sp. (RU47), *Bacillus atrophaeus* (Abi03), and *Trichoderma harzianum* (OMG16)]. The average CFU/mL of the four replications per treatment is presented as mean ± SD.

	Control			+BMc		
	ID	CFU Count CFU/mL (10^4^)	No. Isolates	ID	CFU Count CFU/mL (10^4^)	No. Isolates
MP-Ext	101	7.5	3	117	33.5	17
	102	24.5	7	118	90.5	16
	103	22.0	14	119	38.5	23
	104	18.5	13	120	116.5	22
	Mean	18.13 ± 7.5		Mean	69.75 ± 40.4	
MP-Int	105	4.5	13	121	112.5	14
	106	5.5	11	122	261.5	19
	107	11.0	15	123	109.5	11
	108	11.5	13	124	148.0	20
	Mean	8.13 ± 3.6		Mean	157.88 ± 71.3	
CT-Ext	109	27.5	14	125	122.5	10
	110	25.0	15	126	188.0	16
	111	8.5	10	127	81.5	22
	112	13.5	14	128	124.5	13
	Mean	18.63 ± 9.1		Mean	129.13 ± 44.0	
CT-Int	113	29.5	10	129	222.0	14
	114	28.0	10	130	153.0	9
	115	27.0	9	131	111.0	14
	116	40.5	6	132	86.5	11
	Mean	31.25 ± 6.3		Mean	143.13 ± 59.3	
Total:			177			251

**Table 2:** Total number of *Trichoderma* and non-*Trichoderma* isolates as detected by MALDI-TOF MS in relation to tillage practice [TP: moldboard plough (MP) vs. cultivator tillage (CT)], N-fertilization intensity [NI: intensive N-fertilization intensity with pesticide use (Int) vs. extensive N-fertilization (50%) without fungicide use (Ext)], and inoculation of a consortium (BMc) with beneficial microorganisms [*Pseudomonas* sp. (RU47), *Bacillus atrophaeus* (ABi03), and *Trichoderma harzianum* (OMG16)].

	*Trichoderma* sp. Depending on			Other Taxa Depending on			Total		
Treatment	Total	TP	NI	Total	TP	NI	Total	TP	NI
**Control**									
MP-Ext	14	16	20	23	73	70	37	89	90
MP-Int	2		6	50		81	52		87
CT-Ext	6	10		47	78		53	88	
CT-Int	4			31			35		
**Total**	**26**			**151**			**177**		
**+BMc**									
MP-Ext	53	101	96	25	41	43	78	142	139
MP-Int	48		83	16		29	64		112
CT-Ext	43	78		18	31		61	109	
CT-Int	35			13			48		
**Total**	**179**			**72**			**251**		

**Table 3:** Comparison of estimated duration and costs between the MALDI-TOF- and PCR-based species identification of filamentous fungi.

	Duration			Estimated Cost ^c^ (In Euro)
	Isolation and Culture	Extraction ^a^ (1 Sample)	Data Processing ^b^ (1 Sample)	Per 50 Samples
MALDI-TOF-based method	1–2 weeks	45 min	10–15 min	13.10
PCR-based method	1–2 weeks	45 min	3–4 h (+2–3 days)	484.00

^a^ Duration of protein extraction for MALDI-TOF analysis and DNA extraction for PCR-based methods was estimated based on the number of steps (1 step = 1 min) in the standard procedure and the length of specific tasks, e.g., centrifugation for 10 min, etc. ^b^ Data processing for MALDI-TOF-based methods include loading of samples on polished steel target and reading and comparing protein mass spectra with the Bruker Filamentous Fungi Library. PCR-based methods include PCR amplification and purification, custom sequencing (as additional 2–3 days), and gene sequence analysis, i.e., NCBI BLAST search, construction of phylogenetic tree, etc. ^c^ Estimated costs were computed based on prices of reagents and kits for 50 samples/reactions.

## Data Availability

Not applicable.
